# Challenges in navigating the education-migration pathways, and subjective well-being of highly educated immigrants: the case of Indian student immigrants in the United Kingdom

**DOI:** 10.3389/fsoc.2024.1385664

**Published:** 2024-07-10

**Authors:** Samitha Udayanga

**Affiliations:** Bremen International Graduate School of Social Sciences, University of Bremen, Bremen, Germany

**Keywords:** involuntary immobility, limited capability, misguided aspirations, student migration, subjective well-being

## Abstract

Migration is generally considered to be a driver of enhancing the subjective well-being of immigrants; however, personal characteristics such as educational attainment, migration channel, and country of origin may moderate the impact of immigrant life on expected well-being. Therefore, the present study aimed to explore the intersections between the lived experiences of post-secondary Indian immigrant students in the United Kingdom (UK), the challenges they encounter as immigrants, and how these experiences could impact their subjective well-being. A qualitative research design was employed, which included a focus group discussion and 24 in-depth interviews with postgraduate students who had migrated from India to the United Kingdom. Four themes generated from the thematic analysis, which overall indicated that individuals who came to the United Kingdom as international students to study, work, and settle over time often found themselves unable to leverage their educational credentials to establish expected subjective well-being. This was primarily due to the systematic denial of their agency (capability) to overcome challenges in the host society. Reasons include becoming involuntarily immobile in the host country, incongruency between past expectations and present experiences and prospects, socio-emotional and financial costs associated with immigrant life that hinder the freedom of agency, and bureaucratic burdens. All these reasons can generate an unconducive environment for those immigrants who took part in the study, ultimately decreasing their life satisfaction and positive feelings while increasing negative feelings. In conclusion, the findings question the widely held belief that migration can increase subjective well-being and describe how migration comes at a cost, along with several other challenges, particularly for those who have moved first to learn, secondly to earn, and then to settle in. The channel of migration (migration for education and then to settle in) thus plays a crucial role in determining the outcomes of migration while influencing the anticipated subjective well-being of migrants.

## 1 Introduction

Despite the common perception of migration as a proxy for several positive outcomes, including subjective well-being or happiness, some immigrants struggle to adapt to the norms and customs of their new society, which can hinder the expected overall well-being (Hendriks, 2015; Kóczán, 2016; Hendriks and Bartram, 2019). Several studies indicate that migration initially increases immigrants’ happiness compared to natives (Bartram, 2012; Hendriks, 2015; Brockmann, 2021). This might be because immigrants tend to be healthier, inclined to start a new life, and better educated, allowing them to leverage their capabilities in the host society, in contrast to the lack of opportunities available for them in home countries (Knies et al., 2016; Chen et al., 2022; Zafar and Ammara, 2023). However, the level of initial happiness among immigrants tends to level off over time, and the daily tasks associated with their new lives can become burdensome, leading to decreased life satisfaction (Yaman et al., 2022).

International student migration is a major category of migration, with OECD countries being key destinations for attracting international students (OECD/European Commission, 2023). These students are generally referred to as ‘ideal’ immigrants because of their perceived higher likelihood of better integration into host countries (Krannich and Hunger, 2022). Post-secondary international students are important in several ways, serving as a vital source of labor and contributing to the host country’s economy through tuition and living expenses (Crossman et al., 2020, 2022). Furthermore, many of these immigrant students are channeled into pathways for permanent residency based on their credentials (Hari and Wang-Dufil, 2023). As native populations near retirement age, skilled immigration through student-to-work pathways has been deemed essential in countries that have adopted a two-step immigration framework (Schinnerl and Ellermann, 2023; Weber and Van Mol, 2023). This framework encourages international students to first acquire country-specific credentials and work, and then transition to become permanent residents. The United Kingdom has also adopted a two-step migration process, hosting a large number of international students, the majority of whom are Indian immigrants (Bolton et al., 2023).

Migration often leads to an increase in subjective well-being among immigrants (Helliwell et al., 2018). This is primarily due to the favorable living conditions in the host society compared to their home country (Bartram, 2013). Subjective well-being, which can be broadly defined as a person’s overall life satisfaction and propensity to feel good, serves as an indicator for several positive outcomes (Kahneman et al., 1998). These include health, social relationships, immigrant integration, productivity, and enhanced civic engagement. Studies indicate that international migrants rate their quality of life approximately 9 percent higher after migration (Helliwell et al., 2018). The World Happiness Report 2018 further supports this, showing that international migrants tend to experience 5 percent more positive emotions and 7 percent fewer negative emotions (Helliwell et al., 2018). These findings suggest that migration can enhance subjective well-being by providing individuals with new opportunities and helping them leverage their abilities to improve their quality of life. The extent to which immigrants achieve their objective aspirations, such as better living conditions, also influences their subjective well-being (Hendriks, 2015). The strategies immigrants adopt to integrate into their new host societies play a crucial role in determining their happiness. Furthermore, an immigrant’s past experiences and future prospects can shape their current immigrant life experience. However, these intersections have received little attention in well-being studies (Durayappah, 2011).

Moreover, educated immigrants, or those who have tertiary-level or higher educational qualifications, are five times more likely to migrate and leverage their educational qualifications to establish a better quality of life (OECD/European Commission, 2023). These educated migrants also play a crucial role in many OECD countries, including the United Kingdom, in terms of their contribution to the labor market (Hou, 2024). The two-step immigration policy directly targets these immigrants, enabling them to earn educational qualifications in the host country while working temporarily (Gregory, 2015). They are thus eligible to obtain permanent residency. This makes these immigrants a special category, as most international students migrate to the United Kingdom not solely for education purposes but utilize education pathways as an entry to the United Kingdom, believing it will increase their quality of life, which is believed to be difficult to achieve in their home countries.

While international migration tends to increase happiness for many, some studies also indicate that it can decrease overall well-being in certain circumstances (Mattoo et al., 2008; Huynh et al., 2023). One such circumstance is brain waste, where the host society does not recognize the educational qualifications of international immigrants (Mattoo et al., 2008; OECD/European Commission, 2023). Moreover, immigrants tend to be healthier than natives, some immigrants may experience psychological struggles that can reduce their subjective well-being (Shea and Wong, 2022). Studies show that a significant number of immigrants have traumatic experiences that can lead to mental health problems, which are more pronounced among women (O’Mahony and Donnelly, 2007). Furthermore, factors including economic hardships, limited social connections, and severe weather conditions have been identified as causes for diminished life satisfaction among immigrants (Hendriks, 2015). Despite these potential negative outcomes, research also shows that immigrants tend to utilize their social relations to build resilience in their migrant lives (Alegría et al., 2016). Moreover, involuntary migration, such as for refugees and internally displaced people, presents a contrary case where subjective well-being is often difficult to establish (Hendriks, 2015).

Indian immigrants have a higher level of education than other immigrant groups in several OECD countries (OECD, 2023). Indians have migrated to European countries, primarily for educational, economic, and family purposes (Chand, 2012). While some have returned to India, others have decided to settle in those countries, citing the perceived lack of opportunities for young people in the Indian context (Lindley, 2009). The culture of their home country is one of the main determinants that plays a crucial role in shaping the experiences of immigrants, and it is closely associated with their subjective well-being. Despite the limited studies examining the impact of home country culture on immigrants, some research suggests that immigrants often maintain their home country culture even after assimilating into the host society (Contini and Carrera, 2022; Udayanga et al., 2023). Therefore, culturally formulated goals and perceptions of happiness can sometimes contradict with the cultural norms of the host society (Contini and Carrera, 2022; Andersson and Øverlien, 2023).

Studies have generally focused on the determinants that lead to difficulties in immigrant lives, which can include a lack of social connections, financial issues, cultural differences, and the stigma associated with immigrant lives (Kóczán, 2016; Daru, 2021). What is missing is an understanding of how better educated immigrants make sense of immigrant lives, and how they identify challenges for acquiring expected aspirations while navigating the pathways of *education, work, and settlement trajectory*. On the other hand, studies often focus on the present situation of immigrants’ happiness experiences, largely overlooking the impact of their past experiences and prospects. Therefore, the present study aims to show how Indian immigrant students make sense of their immigrant lives while identifying challenges in establishing their expected quality of life. The findings will provide insights into how Indian immigrant students’ capacity for agency has been curtailed by various factors, preventing them from establishing subjective well-being.

## 2 International student immigrants in the United Kingdom

The United States of America, Canada, the United Kingdom, and Australia are among the most preferred educational destinations, largely due to their status as English-speaking countries (OECD/European Commission, 2023). In recent years, Canada, the United Kingdom, and Australia have received a large number of international students. The United Kingdom has a unique historical position, having hosted educated immigrants for a longer period compared to other countries that primarily received low-educated economic immigrants (Chan and Hurst, 2022; OECD/European Commission, 2023). It continues to be a popular choice for students from India and some former British colonies, drawn by the reputation of its educational institutions and the anticipated higher quality of life in the county (Bolton et al., 2023). Additionally, the two-step immigration process in the United Kingdom, Canada, and Australia specifically attracts many post-secondary students from Asia who plan to settle after graduation (Weber and Van Mol, 2023).

The education levels of immigrants in the United Kingdom are notably high, with over half holding tertiary degrees (OECD/European Commission, 2023). Despite the complex, and extensive nature of emigration, several thousand Asians seek student visas in the United Kingdom (Office for National Statistics, 2023). In 2022, over half a million students were enrolled in UK higher education institutions, primarily from non-EU countries (Bolton et al., 2023). According to OECD data, the UK has consistently been the top destination for many non-EU international student immigrants (OECD/European Commission, 2023). However, the United Kingdom has transformed from being a thriving global hub of revenue-generating international students into a place facing challenges in accommodating an overwhelming number of international students without adequately addressing even their basic needs. Expected home ministry reforms on international immigrants highlight that receiving an overwhelming number of international students can harm both immigrants and natives simultaneously (Duttagupta, 2023). Following the United Kingdom withdrawal from the European Union (Brexit), enrolment of EU students in British institutions has declined significantly. However, there has been an increase in Asian students, including those from India, arriving in the UK to study (Falkingham et al., 2021). In line with this, a new post-study work visa system was introduced in 2021. This system allows those who complete higher degrees to remain in the country and work for a minimum of 2 years (Bolton et al., 2023). After 2 years, these students are required to either return to their home countries or apply for a general work visa. Often, immigrants aim to apply for another visa in order to remain in the United Kingdom.

In recent years, the demographic composition of higher education institutions in the United Kingdom has shifted significantly, with international students now outnumbering domestic students (Bolton et al., 2023). Statistics of the United Kingdom reported a marked decrease in the enrolment of domestic students at Russell Group universities^1^ since 2004 (Bolton et al., 2023). China and India are the leading countries sending students to United Kingdom universities, with long-term student visa holders accounting for 39% of non-EU students in 2023. In that year, India surpassed other nations in the number of sponsored study visas granted, issuing 142,848 to Indian students (Bolton et al., 2023).

## 3 Indian students’ migration to the United Kingdom for higher education and subjective well-being

The desire to study abroad has long been common among many middle-class Indians (Perez-Encinas and Rodriguez-Pomeda, 2021). In India, the relatively high graduate unemployment rate of about 18% has led to an increasing number of Indian students considering migration as a viable option (King and Sondhi, 2018). Despite the high cost of education, studies indicate that the cultural capital gained in reputable United Kingdom institutions motivates many Indian students to pursue international education (Ellis-Petersen, 2023). In addition to their reputation, the limited opportunities in India drive numerous students to seek education abroad, especially for bachelor’s and master’s degrees. Although higher educational opportunities are provided for students in the Indian context, many young Indians are willing to pursue education abroad as a way to leave the country and increase their overall living standards. Women, in particular, are likely to migrate as students, mainly not for education itself, but to settle abroad through education-migration pathways.

As an increasing number of Indian students immigrate to the United Kingdom for higher education, aiming for a higher quality of life, several challenges emerge, including the unaffordable cost of accommodation and tuition fees (Hercog and van de Laar, 2017; Khanal and Gaulee, 2019). Policy reports highlight that, after paying for accommodation, students are left with insufficient funds to meet their basic needs (Bolton et al., 2023). The challenge of finding accommodation is compounded not only by high prices but also by some natives’ belief that the Asian way of life is not suitable for other tenants. According to Daru (2021), the psychological well-being of Indian students in the United Kingdom has declined due to a set of challenges associated with the difficult transition to a new education system, cultural differences, distress, and discriminatory experiences.

While studies are concise in revealing the decline in subjective well-being of Indian students in the United Kingdom, some determining factors for immigrant unhappiness have been recognized. Kóczán (2016) found that acculturative stress can lead to stress and decreased life satisfaction, asserting that immigrants are less happy than natives due to issues related to economic integration and less favorable employment conditions. However, the impact of cultural belonging is considered less significant compared to economic integration. On the contrary, some studies emphasize cultural integration as a main driver that determines the extent to which immigrants’ subjective well-being is ensured (Algan et al., 2013). Additionally, settled immigrants exhibit higher levels of dissatisfaction compared to both natives and temporary migrants in countries like the United Kingdom and Canada. For example, Safi (2010) demonstrates that relative dissatisfaction persists among immigrants long-term, even across generations, though this varies across different ethnic groups. Regarding immigrants in the United Kingdom, Knies et al. (2016) explored that ethnic minorities display lower levels of life satisfaction than the majority of natives across generations. In broad terms, several studies confirmed that migration has brought about positive outcomes, yet some individuals have encountered challenges leading to a decline in life satisfaction.

## 4 Theoretical perspectives

The aspirations-capabilities framework is employed with Bourdieu’s explanation of cultural capital, together with 3P model on subjective well-being in the present study (Bourdieu, 2002; Durayappah, 2011; Carling and Schewel, 2018). The aspirations-capabilities approach suggests that a person’s capabilities and aspirations to migrate allow capable individuals to migrate by leveraging their capabilities. (Carling and Schewel, 2018).

Migration aspirations are a function of people’s general life goals and perceived prospects for life (Carling and Schewel, 2018). Capabilities are contingent on positive and negative liberties. Thus, freedom is also a vital ingredient in migration aspirations, particularly for educated migrants who decide to migrate, thinking of better opportunities elsewhere, despite relatively fair living conditions (Carling and Schewel, 2018; de Haas, 2021). These people aspire to enhance life satisfaction furthermore with the help of their educational credentials, and hence their migration experiences might be different from those of other migrants, such as economic or family migrants. Highly educated migrants seem to migrate not solely for educational purposes but, as research suggests, for establishing honor or prestige within their families (Bhugra et al., 2020). This is particularly true for Indian immigrants, as the present study shows. Therefore, the cultural capital theory is also an important perspective employed in analyzing the data (Erel, 2010).

Subjective well-being generally consists of two dimensions: evaluative and experienced aspects. The evaluative dimension refers to how an individual evaluates or rates their level of happiness, while the experienced dimension shows their experiences with happiness (Durayappah, 2011). Experienced subjective well-being is important for the present research, as it delves deep into understanding the experiences of immigrants. Furthermore, the 3P model of subjective well-being is also at the center of the present study. Subjective well-being is a difficult concept to define, yet Bryant (2003) suggested that it is the capacity to regulate contentment, find it, manipulate it, and sustain it. Therefore, subjective well-being is also a process associated with everyday life. The 3P model (Past, Present, Prospects) captures this and indicates that past experiences, present situations, and future aspirations of people are equally important for determining subjective well-being. Subjective well-being can consist of several dimensions, but mainly it includes life satisfaction, positive feelings, and low negative feelings. Therefore, subjective well-being is not apparently manifest, yet it is a result of several other determinants. Those determinants can include feeling good in life, satisfaction with life, freedom of choice, having healthy relationships, and a few chronic worries (Durayappah, 2011). However, these conditions should be understood in relation to past experiences and prospects. With regard to highly educated migrants, past experiences associated with migration aspirations and future expectations of migration would be extremely important, because their migration intentions are often pre planned and well informed.

**Figure 1:**
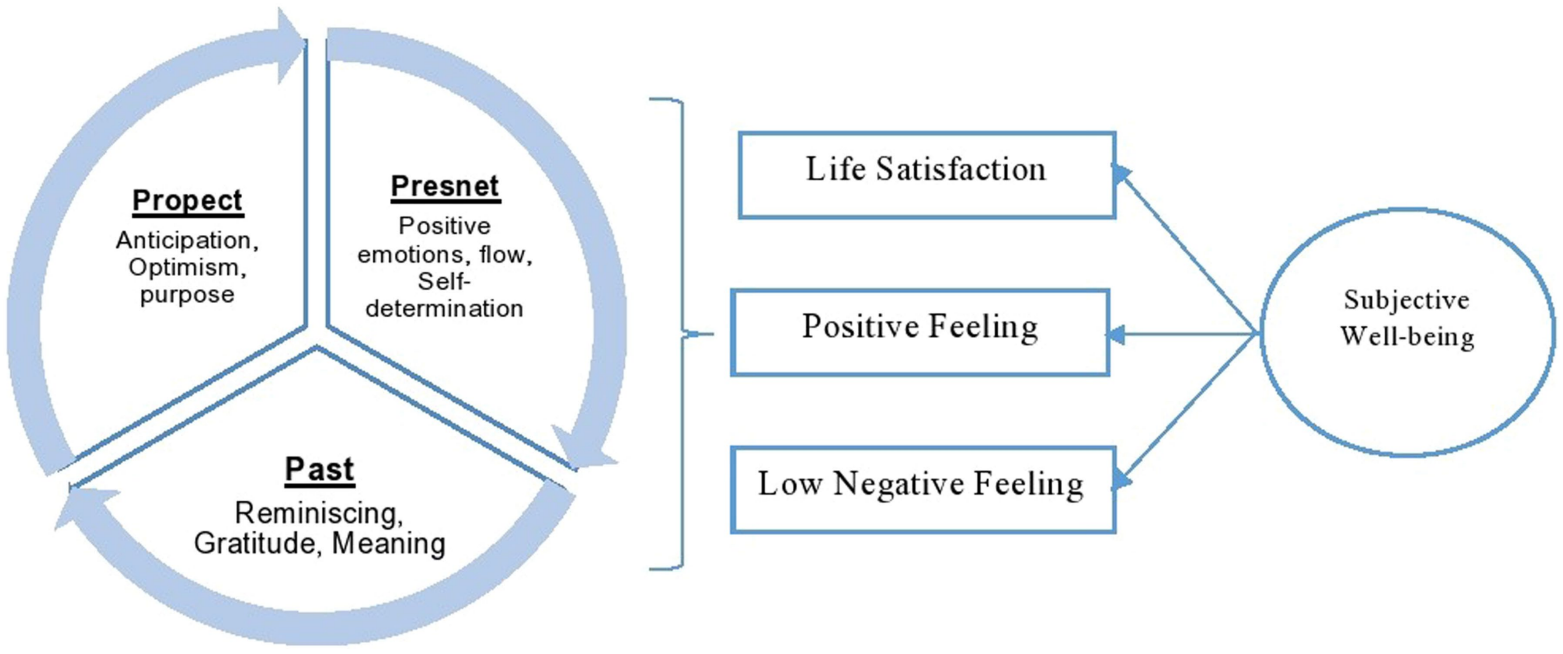
Constructed in line with the 3P model of the subjective well-being (Durayappah, 2011).

Even though several studies on the subjective well-being of immigrants have been conducted as highlighted above, how past experiences, present challenges, and future expectations interact together in determining or challenging subjective well-being has not been well understood. Therefore, the use of the 3P model (past, present, and prospects) in this qualitative study would reveal how migration trajectories are associated with establishing subjective well-being and hindrances to it (Figure 1). This 3P model is important because it utilizes the time aspect of subjective well-being, which was not observed with international immigrant students. Several dimensions that can determine life satisfaction, positive feelings, and low negative feelings are associated with past, present, and future dimensions (Durayappah, 2011). The challenges to establishing subjective well-being should thus be understood in line with these dimensions.

## 5 Methods

The study looked into how life satisfaction, positive feelings, and low negativity can be disturbed when they navigate their migration trajectories, with a focus on the temporal dimension. Since most of these reasons are unexplored, a qualitative research approach was employed to explore the factors affecting immigrant trajectories (Bryman, 2008; Zapata-Barrero and Yalaz, 2022). Relying on a constructivist ontology, the study aimed to understand how immigrants comprehend their immigration journeys in relation to challenges encountered in establishing their expected aspirations (Crotty, 1998; Bryman, 2008). The research involved one focus group discussion and twenty-four in-depth interviews with a group of purposively selected participants.

The participants of the study included Indian immigrant students who came to the United Kingdom mainly through a two-step immigration process: first for study and then to settle long-term. The recruitment criteria included having stayed in the United Kingdom for at least a year, intended to settle in the country, and enrolled in postgraduate degree programs. There are some participants who have completed their postgraduate education and entered the labor force. Participant recruitment was carried out through their social media groups, as almost every immigrant is part of such groups (Stern et al., 2017). Social media groups (such as Facebook groups of immigrants) are often used as a means to share their experiences and information with regard to opportunities in the United Kingdom. These social media groups facilitate interactions among immigrants from diverse regions across the United Kingdom. While describing the purpose of the study, participants who matched the recruitment criteria were invited to interviews, who were first selected from a Facebook group which consisted of more than 50,000 Indian students. Guided by the initial participants, further participants were chosen until the data saturation point was met. Reflective debriefing was conducted following each interview session to gain an initial understanding of the experiences as articulated by participants. This was important for capturing the data saturation point when participants do not contribute to revealing further new experiences (Saunders et al., 2018). In total, 24 participants took part in in-depth interviews. Four participants had already settled in the United Kingdom; six had started the application process; and the other 14 had just arrived in the United Kingdom and were still studying or working after graduation. All participants were over 25 years old and under 40 years old. There were 15 men and 9 women among the participants. Eighteen were married or in a legal relationship, and the others were single. Among married persons 10 were parents. Four participants were enrolled in PhD courses, and the others were enrolled in master’s-level courses.

Firstly, a focus group discussion (FGD) was conducted with five participants to gain an understanding of how Indian immigrant students perceive life in the United Kingdom, the challenges they encounter, and how it impacts their well-being. Three men and two women participated in the FGD. The focus group provided a broad overview of the challenges experienced by Indian immigrant students, those who immigrated primarily for postgraduate studies. In the FGD, participants discussed some commonly experienced challenges in the education-migration pathway. Secondly, in-depth interviews were conducted using a semi-structured questionnaire to understand challenges encountered by immigrant students that could decline their subjective well-being. All interviews were conducted in English, adhering to the ethics approved by the institution. All interviews and the FDG were conducted online, and interviews were recorded. All participants provided informed consent, ensuring that their participation was voluntary.

Recorded interviews were transcribed and analyzed thematically using MAXQDA (2020) (Braun and Clarke, 2006). First, the nature of the data was understood through focused reading and familiarized with the narratives. Then, a code set was developed using both *in-vivo* codes and theoretical codes. The author of the study, accompanied by research assistants during the data collection process, participated in the coding phase. The codes were combined into themes, which were then reviewed and revised to create a coherent story. Overall, four themes, combined with a set of sub-themes, were generated that show how Indian student immigrants in the United Kingdom identify challenges when they navigate the education, work and settling in trajectories which can decline their subjective well-being. Throughout the research, attention was given to quality criteria to ensure the trustworthiness of the research (Lincoln and Guba, 1985).

## 6 Results

**Figure 2:**
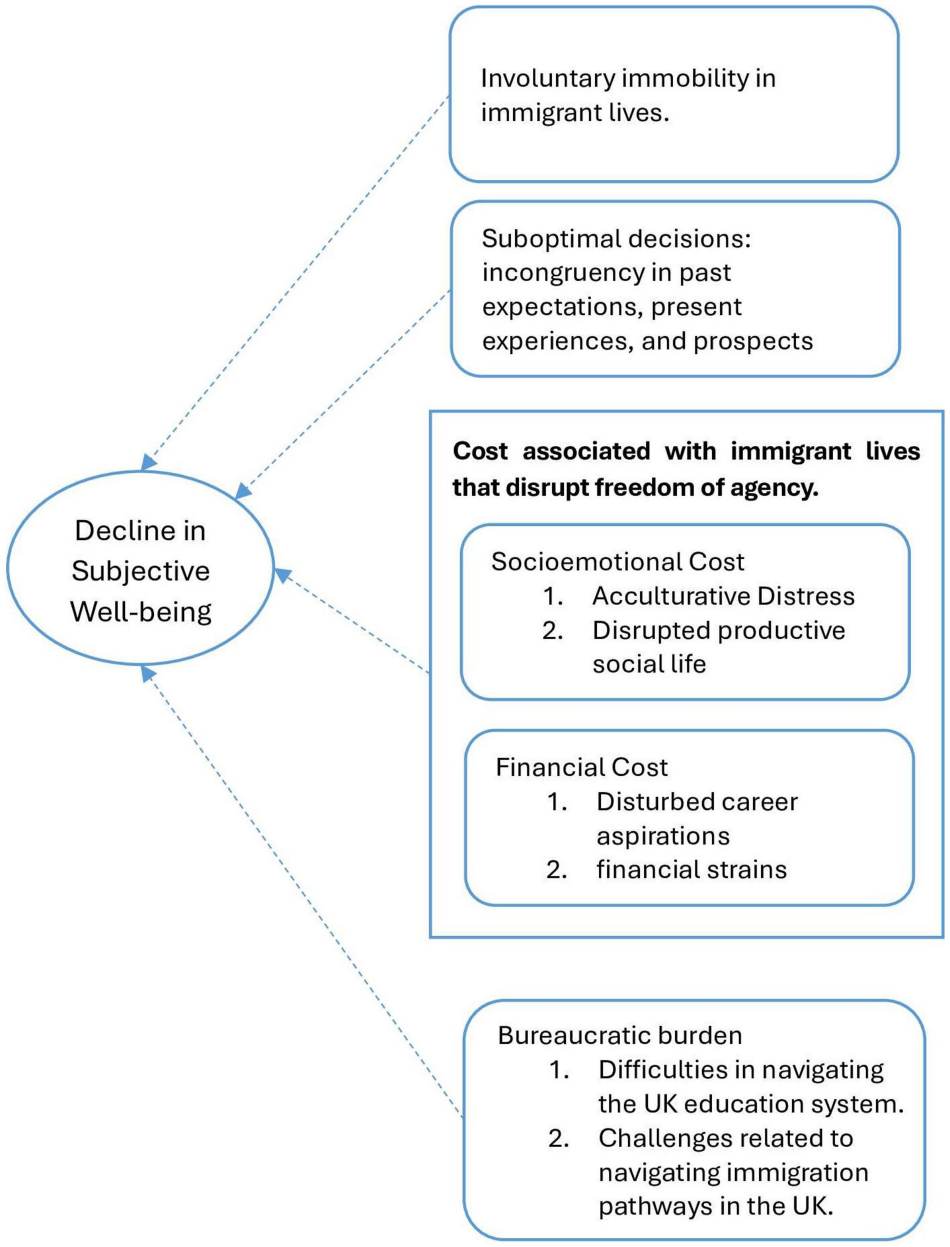
Thematic map.

Figure 2 illustrates the thematic map generated during the analysis. It broadly shows the four themes generated, and how each of the theme connected to decline in subjective well-being of highly educated Indian immigrant students in the United Kingdom.

### 6.1 Theme 1: involuntary immobility of immigrants

This theme shows how Indian student immigrants have become trapped in immigrant lives, deviating from the capability (freedom) to regulate their lives, navigate challenges effectively, find what they value for their lives, and sustain those endeavors to establish their subjective well-being. This theme identifies capability or freedom of agency in migrant lives as a core pillar of subjective well-being. However, a number of factors deprive Indian student immigrants of this freedom, causing them to become involuntarily immobile or trapped, disrupting their current lives, and potentially leading to unsettling future aspirations.

Respondents describe that “they feel stuck in the United Kingdom.” Although they immigrated voluntarily with certain educational credentials, the analysis shows that they feel “stuck” in the United Kingdom, left to face the even more unbearable challenges of immigrant life alone, mainly when trying to balance studies, work, and family responsibilities. They have come to the United Kingdom with an informed decision, and as a result, the expectations they had developed before migration were very high. Those who migrated are often middle-class immigrants, and they had a fairly good socioeconomic background in their home country. However, now unexpectedly they encounter difficulties that undermine their way of life. This altogether makes them stuck in the immigrant lives.

“I came to the UK thinking that I could elevate my and my family's quality of life further. However, now I am unable to bear the burden, which I did not expect. In this situation, I do not think that I can envision a better future”. (Male, 31-years-old)

“Living in the UK is difficult. I did not expect such a situation before departure. Everything is expensive; my income cannot cover that. At the same time, my children are getting used to the UK, and they do not wish to return. But I feel like going back, so now I feel stuck in the UK. I have no choice until they can manage themselves as adults. I would love to go back home!” (Male, 39-years-old)

Although life in the United Kingdom may appear more challenging than life in India, immigrants make effort to remain owing to two main reasons. Children who have been raised in the United Kingdom would probably find it difficult to adjust back to India. Furthermore, returning home would engender sentiments of disgrace among the immigrants themselves and their family members. As a result, immigrants tend to stay ‘somehow’ at least for a certain period, and this deviate them from the freedom of life. Despite their educational credentials, they seem to have deviated from individual agency to navigate the host society effectively. This leads to a decline in life satisfaction and an increase in negative feelings. In this regard, the temporal comparison is significant, as immigrants tend to compare their present situation to their past lives and the expectations they developed before migration. At the same time, the present instability does not allow them to develop positive feelings about future aspirations.

“We find ourselves practically trapped in the UK due to substantial debts incurred for visa, migration, and tuition fees. There is no possibility of saving until both of us work, balance our studies, and provide support to our family back in India.” (Male, 30-years-old)

Students who immigrated with dependents are often overburdened with family responsibilities in addition to study and work obligations. Unaffordable accommodation and high living costs are major concerns for these immigrants. While earning and learning, they are also obliged to financially support families back in India. Having invested heavily in migration preparation, the migration process, and educational institutions in the United Kingdom, they feel stuck without any choice but to work and stabilize their immigrant lives. Beyond expectations, a considerable amount of time has to be allocated to earning enough to repay debts, pay tuition, and cover everyday living expenses. As a result, as they describe, there is no time left for essentials like adequate sleep, leisure, social gatherings, or life enjoyment.

Another participant shows how difficult it is for a newcomer to settle in and that migration comes at an unbearable cost, which is also difficult to compensate for.

“Life is truly difficult for all newcomers. Especially, finding a job is extremely difficult for us with no experience. Believe it or not, the UK is not a place for newcomers. You have a lot to lose.” (Female, 25-years-old)

One main reason driving the emigration of Indians to the United Kingdom is the belief that migrating will give them prestige and honor. Indians believe the United Kingdom to be a good country for education and living. However, once immigrated as students with the expectation of settling long-term, immigrants are likely to realize they have to lose many things just to become immigrants. Therefore, feelings of wanting to immediately return emerge among participants, along with regret over the decision to migrate. The causes of these sentiments will be explored in the third theme. Overall, the regret leads to decline in subjective well-being as they strive to navigate the host society environment.

“I am planning to return to Gujarat, India. I regret wasting years of my life studying here. Life could be better in India. It is better to stay here for five to ten years, maximum, if you want to be financially settled, and then move back.” (Female, 38-years-old)

“I wish I could leave this place and live a simple, stress-free life surrounded by family and friends.” (Male, 29-years-old)

Individuals who initially arrived as students and aspired to settle in the United Kingdom for the long term seem to regret their decision. The unexpectedly higher cost of living in the United Kingdom has led them to realize the value of the simpler way of life in India, surrounded by family members as above narratives explain. This is where they strive to associate objective well-being with subjective well-being, that can altogether lead to a life satisfaction. Those who have stayed in the United Kingdom for a considerable period also express that their lives might have been better in India, where they could have maintained strong social connections with family and friends.

The shift in how Indian immigrant students define “overall well-being” is also an important determinant that can contribute to the decline of positive feelings. Before migration, Indian migrants perceived expected happiness as encompassing well-paid jobs and a luxurious and liberal lifestyle. However, the data indicates that post-migration, these immigrants tend to reevaluate the significance of family connections, a favorable climate, the Indian way of life, and a simple lifestyle as fundamental components of subjective well-being. The contrast between their past and present lives leads them to realize that their current circumstances fall short of their expectations prior to emigration.

“Parents find themselves caught in a dilemma, attempting to provide their children with a foreign education while also inculcating Indian values in them.” (Female, 31-years-old)

“We are unable to secure jobs here, which is unfair, especially considering that we have mortgaged houses. Consequently, students like us are compelled to earn more and more to alleviate existing loan obligations. We must earn money for our survival and send funds back home to repay debts.” (Male, 29-years-old)

At the same time, the stigma associated with an immediate and unsuccessful return can compel individuals to endure suffering while staying in the United Kingdom. India, being a society with a strong culture of honor, closely associates foreign education with prestige. Consequently, immigrant students feel a moral obligation to their parents to complete their educational journeys or *somehow* settle in the United Kingdom. Failure to do so might jeopardize the honor gained through the migration. Therefore, the stigma associated with an incomplete return forces students to bear the consequences of unexpected issues linked to the immigration trajectories they navigate as students, workers, or members of their families. A student explains:

“I really want to go back to India now. But if I do so, my parents will be saddened. How would they face the community? I know of a case when a student like us returned to India without success in the UK, and the mother of that student committed suicide because of the shame she felt from other women in the community. I do not want such a result.” (Women, 35-years-old)

At the outset of their journey toward becoming permanent residents in the United Kingdom, participants are likely to feel unhappy. Yet at the same time, they think this will gradually level off, and they will gain the freedom to exercise agency to overcome barriers and establish expected aspirations. However, some participants describe this as a happiness trap. After some years of immigrant experiences, they reflect that the past few years have been a waste compared to the life they would otherwise live in India. This also shows that life before migration can have a significant impact on immigrant lives, in that those who migrated with the intention of fleeing a country with limited opportunities might somehow tend to stay stronger, compared to those who have migrated with a fairly good socioeconomic background. Yet in the present study, sufficient data is not available to develop this claim.

“I lost my part-time job and was dead broke. But I persevered through the cold and harsh weather, completed my studies, got a job, saved money, and set up a firm. This all happened in thirteen years. The UK is not for the weak. After all these years, I am now thinking of going back to India, as I believe this is a constant struggle focused on some future happiness, which is never fully attained.” (Male, 40-years-old)

Similar to this respondent, many other newly emigrated students believe their unhappy struggles during the initial stages of immigrant life will pay off with expected future happiness. Although they envision a better life in India, the prospect of eventual happiness in the United Kingdom motivates them to stay, even while engaging in tasks that likely would not lead to life satisfaction.

### 6.2 Theme 2: suboptimal life aspirations: incongruency between past expectations and present experiences

Mainly, Indians anticipate a better and happier future in the United Kingdom, only to realize that their actual experiences after migration fall short of expectations. The acquisition of a student visa is often promoted as an easy entry point to the United Kingdom. However, students, along with their parents, are often misled about the abundant opportunities supposedly accessible in the United Kingdom. This misinformation is primarily propagated by agents facilitating foreign education and even through social media channels. Unattainable aspirations associated with a luxurious foreign lifestyle are also promoted through the social media engagements of young potential student migrants in India. Comparisons between their current lives and the celebrated lives of their foreign peers, exposed on social media, shape their migration aspirations. As a result, several students are moving to the United Kingdom with the long-term goal of settling through educational pathways, often without realistic educational objectives. The lack of genuine motivation for foreign education, coupled with the obligation to complete education as a requirement for visa acquisition and for the long-term aspiration of settling in, can generate several unexpected challenges.

“I met several Indian students who were unclear about their goals because they did not have proper guidance. Many of them wanted to earn money while getting a UK degree. Some even thought they could earn enough to cover their living expenses.” (Male, 30-years-old)

“I thought it would be simple, like money growing on trees and jobs being easy to find. That is what some of my friends and even agencies told me.” (Female, 32-years-old, FGD)

Prior to migration, several participants often lacked a comprehensive understanding of the post-migration scenario, thereby fostering unrealistic expectations. Therefore, there was an incongruency between what was expected before migration and what was really experienced after migration. The aspirations they had pre-migration have become a fallacy in the United Kingdom for several immigrant students, as they often failed to fulfill them.

“We lack work experience, and when faced with employers, they often request prior experience. We were misled into believing that jobs are readily available and easy.” (Male, 31-years-old, FGD)

“Often, students refrain from pursuing jobs beyond the first day due to their perceived difficulty. Only a handful of students secure jobs that bring satisfaction. A majority lack the necessary experience and qualifications. However, they were misled into expecting an abundance of satisfactory job opportunities in the UK.” (Male, 40-years-old, FGD)

This indicates that student immigrants lack a clear understanding of the United Kingdom job market and are frequently misinformed. The analysis also reveals that individuals who have previously immigrated are not often forthcoming about the reality to newcomers. As a result, even when newcomers explore social media and other websites, they are often presented with an idyllic life filled with happiness, lacking authentic information about the actual lifestyle after immigration. Brain waste is also closely associated with misguided aspirations. Highly educated migrants migrate mainly to leverage their education and benefit from it. However, the data shows that in addition to the difficulty in accessing the labor force, available employment does not match the educational credentials of immigrant students. Oftentimes, they are unable to benefit from their higher educational qualifications. Despite their heavy investment in education, current immigrant students also believe they will not reap any favorable returns in the future. This shows how their expectation before migration is not aligned with the current experiences and prospects. For example:

“I had a BA degree and came here to pursue a master’s degree in social sciences. Now I work in an elderly care home. Back in India, I would have benefited from my education, but now there is no relationship between my educational qualifications and what I do in the elder care home”. (Female, 39-years-old)

Furthermore, student-sending agencies play a key role in the misleading process. These agencies encourage Indian students to pursue education abroad, suggesting that obtaining a student visa is an easy entry point to the United Kingdom. The motivation among Indians to migrate as students has been captured as a market strategy by those students sending agencies. Such advice creates a pre-departure burden among immigrants who must secure financial resources, apply to educational institutions, and prepare for IELTS examinations, among many other pre-departure activities.

“Agencies promise top-notch education and easy job opportunities, but the reality is different. In my class, most students are from Africa or South Asia, and there are few British students.” (Male, 26-years-old)

The suboptimal life aspirations developed before departure negatively affect the life satisfaction among participants. Although no particular attention has been given in the present paper, it is clear that those who have a realistic idea about post-migration experiences and have social networks in the United Kingdom, such as family ties or friends, can more effectively navigate the problems they encounter and establish life satisfaction.

### 6.3 Theme 3: cost associated with immigrant lives that disrupts freedom of agency

A successful immigration life is characterized by the capability or freedom to feel positive in life, regulate lives in a way that leads to life satisfaction, and sustain it. However, the findings in the present study show that participants bear a heavy cost in maintaining immigrant lives, including emotional and social costs in addition to the commonly known financial cost. Despite possessing higher levels of education, immigrants encounter challenges in leading a satisfactory life. Beyond the several known factors that affect their ability to function effectively, this paper presents two main drivers that can hijack immigrants’ capability to establish life satisfaction. Namely, socioemotional cost and financial cost. While participants frequently identified weather-related depression and difficulties accessing healthcare as potential drivers that can hinder a conducive environment for immigrants, the present analyses did not include these factors, as several previous studies have already focused on those aspects.

#### 6.3.1 Socioemotional cost: acculturative distress

Acculturative distress, a common experience among immigrants, refers to the psychological and emotional challenges they face when adapting to a new culture. Acculturative distress is linked to commonly known factors, such as being detached from the family environment in India and experiencing a mismatch in values, among several others.

“After I got a job, I spent most of my time with friends from Europe, where I learned about their culture and way of life. Even though they look happy, I think they are missing something. Maybe it is a spiritual connection with each other. And I made up my mind that I would never have a family here. India is where I want my kids to live.” (Male, 35-years-old)

In addition, participants in the present study strongly highlight the intergenerational cultural difference that can negatively affect Indian student immigrants, who aspire to live an Indian way of family life in the United Kingdom.

Considering future family life and children, participants illustrate the belief that the current United Kingdom culture would not be suitable for an ideal family life. The anticipated family life in the United Kingdom does not align with the expectations developed before departure. The Indian way of life is deeply ingrained in their existence, and adapting to a new cultural life elsewhere is likely to pose challenges for them.

“The UK is not a good place for kids to live with their parents. We can’t expect our kids to treat us the way we treat our parents. The UK will be very lonely for us in twenty to twenty-five years.” (Female, 31-years-old)

Even though Indian immigrants come to the United Kingdom partly due to the lack of opportunities in India, they strive to hold on to the cultural values instilled in them even as immigrants. Data suggests that immigrants fear for their future, as they perceive that their children will not be ‘ideal children’, those who are more adapted to Indian culture.

Furthermore, acculturative distress arises as a result of occasional discrimination and racism.

“When I was riding the tram at night, a group of students approached and sat around me. A girl in a fancy dress sat in front of me, kept her leg on my bag, and said bad things to me. I felt really bad and got off at the next stop.” (Female, 35-years-old)

Participants often note an outsider feeling stemming from their non-British origins, which often leads to regret. Regardless of the achievements they may attain, there is a persistent belief that they would be happier with family and friends in India.

#### 6.3.2 Socioemotional cost: disrupted productive social life

When students are overburdened with study and/or work, their social life is likely to suffer. Originating from a culture with strong social ties and vibrant social lives, loneliness and the stress of balancing work and education diminish the anticipated free and happy life for Indian immigrant students in the study.

“When individuals are occupied with work, education, and additional responsibilities, they may find it challenging to socialise with others. Additionally, we are not accustomed to mingling with natives.” (Male, 29-years-old)

As every immigrant is tied up with more work and even fulfilling obligations to their home country, they are likely to be devoid of a social life. This is also associated with a lack of support from Indian communities in the United Kingdom, which are restrictive and mirror the hierarchical culture in India. For example, North Indian groups often do not include South Indians. While some are ready to help newcomers, data suggests that Indian communities generally provide a lack of support for new Indian immigrants.

“We Indians are to blame. Sometimes, even here, Indians discriminate by treating North Indians as Vadakans (North Indians) and them calling Madrasis and so on”. (Male, 33-years-old, FGD)

This illustrates how Indian cultural norms are reproduced in the United Kingdom and impact newcomers, deviating them from productive social life. The absence of social ties and support from the diaspora can also exacerbate loneliness and emotional distress for those who are already struggling to make ends meet.

“This life is dull and lonely. If I were to stay beyond the age of 50, I would end up dying in a nursing home without family support.” (Male, 31-years-old)

“India has life that the UK does not have. It is really cold in the UK. That food is not very good. I really missed how friendly my own people were.” (Male, 34-years-old)

In the long term, people fear staying in elder care homes, which is unexpected. The anticipated loneliness and the comparison with the alternative situation in India will foster feelings of loneliness, ultimately leading to decline in positive feeling in immigrant lives.

#### 6.3.3 Financial cost: disturbed career aspirations and financial strains

Indian immigrant students also encounter challenges in reaching their career objectives and experience financial difficulties. Despite arriving in the United Kingdom with the expectation of studying and working, they soon realize the difficulty of finding a suitable job due to a lack of relevant experience. At the same time, they are unable to find an employment which mirror their educational credentials.

“Getting a job is not easy. Many of us work in restaurants, sometimes as room boys or cashiers.” (Male, 27-years-old)

Individuals raised in middle-class families are now oftentimes facing challenges in securing decent employment due to a lack of experience. As a result, they take on any available work to meet their daily needs. Importantly, even after graduation, the data reveals that their educational qualifications do not necessarily translate into improved outcomes in the labor market. This is especially evident for women who may be confined to their homes (with care obligations) or face limited opportunities from employers.

“I did not come here to pursue a career in care work. Like me, many individuals with degrees in business administration or social science end up joining the field of care work, particularly in eldercare homes.” (Male, 30-years-old)

Because educational credentials are not effectively translated into improved labor market outcomes, participants are dissatisfied with their current lives. Students are also sometimes underpaid and exploited. Due to the lack of job opportunities, they tend to work wherever they find the chance, often juggling three to four temporary jobs per day without sufficient time for education, family, or personal enjoyment.

“I work at a post office during the day and then do shifts at an elderly care facility. Additionally, when possible, I take on work as a hotel cashier. This leaves me with little time for education and other activities.” (Female, 32-years-old)

All of this indicates that career aspirations go unmet, leaving immigrant students in a situation where they are unable to leverage their educational qualification to establish life satisfaction.

### 6.4 Theme 4: bureaucratic burden

**Figure 3:**
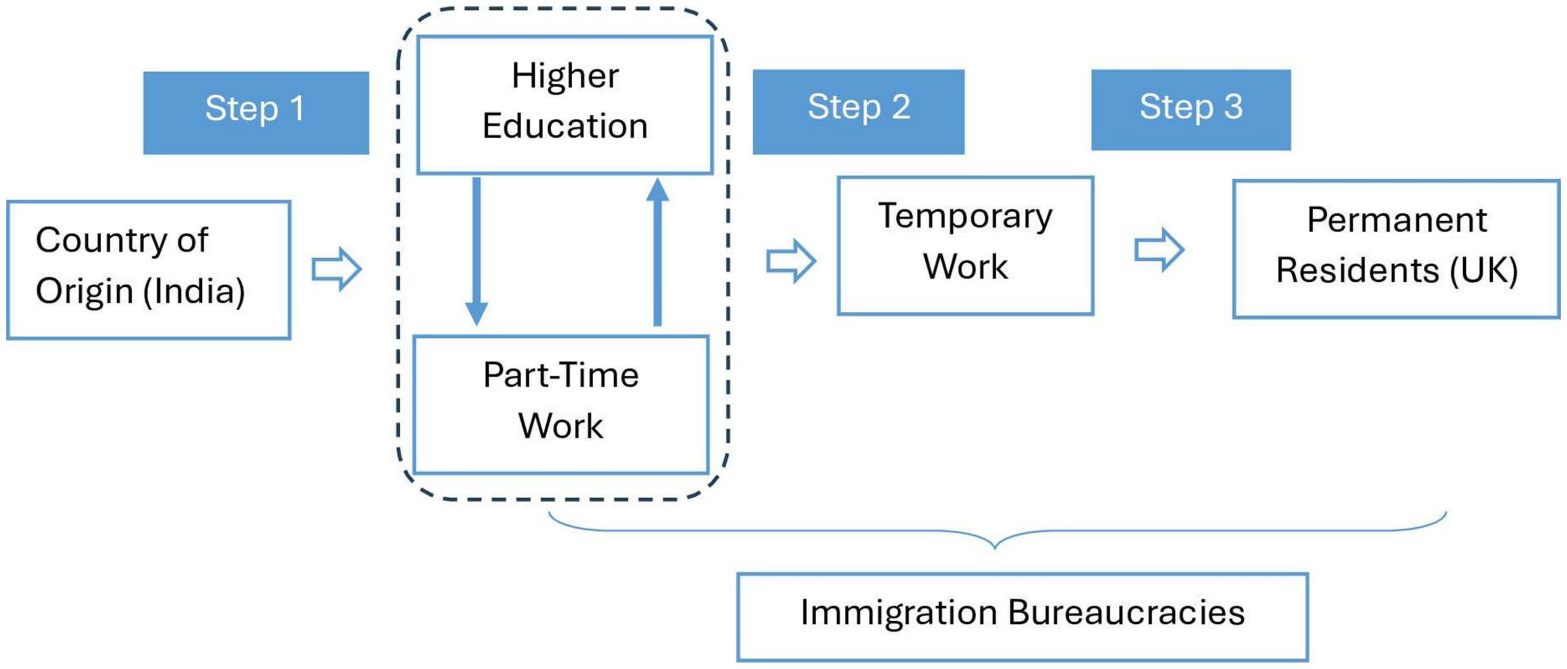
Three step migration process (in line with Brunner, 2021).

Two-step migration has become a three-step migration process which is now the primary structure upon which the lives of Indian immigrant students rely, and they must navigate this system successfully to become permanent residents. This process primarily involves completing higher education, securing employment, and later entering the labor force permanently through a shift in visa categories (Figure 3). Therefore, successful educational achievements and effective negotiations with immigration bureaucracies are central for these highly educated immigrants, compared to other immigrant categories. After graduation, they can enter the labor force for 2 years, but after that, they are required to change their visa type and fully enter the labor force if they are to become permanent residents over time. Although the process of integration is well-articulated, navigating it has become a burden for several participants. As a result, challenges related to education and dealing with immigration authorities have had a severe impact on the experienced well-being of these immigrants.

#### 6.4.1 Difficulties in navigating the United Kingdom education system

Almost every participant emphasized that the United Kingdom education system is not fair enough for immigrants due to extremely high tuition fees. Additionally, in comparison to Russell Group universities, education at other institutions is perceived to be of low quality and overloaded with international students. Meeting the expected educational aspirations thus appears to be challenging.

“The visa for foreign students is a big business for universities, and it also gives employers cheap labour. Some universities purposely fail students in courses to make more money in fees when the students have to retake the exams.” (Male, 35-years-old)

“There, it was like a foreign country with thousands of students from South Asia and Africa, and there were very few white British.” (Female, 32-years-old)

Because United Kingdom higher education is extremely expensive for international students, student immigrants struggle to afford the tuition fees, prioritizing them over other life necessities. The anticipated life in the United Kingdom is thus difficult to realize. Additionally, some students face challenges within the United Kingdom education system, often due to a lack of the necessary preparation. Simultaneously, due to a lack of time and dedication for studying, several students tend to fail continuously and eventually drop out. Therefore, the immigrants’ endeavors toward establishing a satisfactory life have become a burden, which is contrast to what they have anticipated.

#### 6.4.2 Challenges related to navigating immigration pathways

Navigating the policies declared by immigration bureaucracies and fulfilling various requirements until they become permanent residents in the United Kingdom is found to be extremely difficult and an additional burden over existing difficulties. Interactions with immigrant-administering authorities are noted as a severe burden for immigrants, which is also unexpected. Among several challenges encountered during when they navigate visa pathways, finding a sponsorship has been noted as a prominent challenge. Finding sponsorship to extend their visa after graduation is often seen as a challenging policy requirement for Indian immigrant students.

“Many companies can provide a job but are unable to offer sponsorship^2^ to employees because they have not completed the licencing process with the Home Office.” (Male, 40-years-old)

“Nothing comes easily in the UK. You have to work hard and prove yourself in your field. Sponsorships are readily available for those who perform well with the required skills.” (Female, 39-years-old)

Students find it challenging to secure sponsorship, which is necessary for applying for a visa extension. Furthermore, participants acknowledge several bureaucratic challenges, including difficulties in approaching officers at immigration authorities and processing applications. In order to be integrated and have a happy life in the United Kingdom, those immigrants now are required to deal with immigration bureaucracies. This process of navigating the two-step migration system has become an additional burden alongside studying, working, and supporting families, which altogether depreciates the experienced subjective well-being of these immigrants.

## 7 Discussion and conclusion

The present study considers international students as a unique group of immigrants whose migration trajectories are distinct from others, and they may draw on different sources to establish their subjective well-being. Theoretically, it is argued that past experiences can have an effect on the present immigrant experiences and prospects (Durayappah, 2011). This indicates that the challenges highly educated immigrant might encounter in establishing subjective well-being might also differ from other immigrant groups. This was the main focus of the present study, which illustrated how international student immigrants make sense of their immigrant lives and identify challenges in navigating the education-migration pathways. The findings highlight how challenges that limit immigrants’ ability to exercise their agency to make informed choices can impede their overall sense of well-being.

The findings show that four broad domains of reasons can lead to a decline in the subjective well-being of Indian student immigrants in the United Kingdom. The past experiences related to the country of origin and the migration decisions have a strong impact on the present immigrant experiences and the prospect of immigrant lives. Indian students perceive themselves as stuck in the United Kingdom, and their immigrant lives have become an additional burden on top of the challenges of navigating education, work, and settling-in. Furthermore, the incongruence between past experiences, expectations, present life circumstances, and future aspirations creates instability in migrant lives, which harms positive feelings and life satisfaction. The inability of student immigrants to exercise agency in effectively navigating the education-migration pathways contributes significantly to the decline in subjective well-being. The results also indicate that unexpected bureaucratic burdens can negatively affect the lives of immigrants who strive to settle in the country through a two-step migration process, often associated with international students. The reasons outlined in the present study are unique to Indian student immigrants in the United Kingdom because the home country’s culture is a vital ingredient in determining migration aspirations and present immigrant experiences (Contini and Carrera, 2022). The incongruence of migration decisions, present experiences, and disturbed future aspirations is at the center of all the challenges these immigrants experience.

Immigrants with higher levels of educational credentials and skills are also believed to be happier and more satisfied with their lives (Chen et al., 2022), yet the findings of the present study show that several determinants can contribute to depreciating subjective well-being, even in the early stages of migration. While several studies have focused on happiness among immigrants and determinants of subjective well-being, the present study illustrates that the drivers hindering subjective well-being for immigrants are likely to differ from simply being the negation of those positive determinants. Previous studies has identified acculturative distress, forecasting bias, and the unfavorable environment of the host society as factors contributing to decreased happiness among immigrants (Hendriks, 2015; Kóczán, 2016). However, those reasons cannot be generalized for all immigrants, as their personal characteristics, such as their level of education, have the capacity to moderate the effects of those determinants. For example, participants in the present study implied that even though they have a higher level of education, they are unable to leverage it in order to benefit from migration due to their higher levels of aspirations associated with higher levels of education. This is the main reason why they feel stuck in the country, potentially leading to increased negative feelings in their immigrant lives. Yaman et al. (2022) found that deteriorating health conditions can contribute to decreasing levels of happiness. However, for highly educated immigrants, navigating the education-migration pathway has become challenging, and ‘immigrant life’ has become a burden in fulfilling aspirations developed even before migration, as the findings imply.

Migration aspirations play a key role in determining the success of emigration and effective integration in the host society, as aspirations determine the extent to which host society facilitation can be appropriated and benefit from the migration (Borselli and Van Meijl, 2021). Graham and Markowitz (2011) demonstrated that aspirations related to obtaining higher objective well-being are not necessarily associated with life satisfaction; the feeling of achieving aspirations is vital for immigrants’ overall happiness. However, findings of the present research question the common belief that immigrants are happier at the beginning. For highly educated Indian immigrants participated in the present study who have migrated particularly for education, work, and later to settle down, happiness has become a hardly to reach goal even at the very beginning of their immigrant lives.

In addition, how aspirations are shaped before migration would determine the happiness of immigrants. Some have identified that forecasting bias would lead to unhappiness (Kahneman et al., 1998). However, the findings indicate that the unhappiness of immigrants is caused because immigrants were misguided and promoted to develop unrealistic aspirations in the host country, which prove difficult to achieve once they have immigrated. Kahneman (2011) also found that such suboptimal decisions can lead to challenges in the host society, such as barriers to accessing the labor market. The participants in the present study, on the other hand, made suboptimal decisions, primarily because they were misguided and did not conduct adequate prior research on the United Kingdom before departure. The “happiness pull” is thus artificially created among Indian immigrant students, oftentimes without a real picture of the United Kingdom context, and the relative “unhappy push” from their home country is also systematically exacerbated through social media.

Indian student immigrants and their parents back in India consider United Kingdom education to be a cultural capital that can elevate their honor. This is why, despite several difficulties in the United Kingdom, student migrants strive to bear the burden of becoming immigrants until they achieve their future aspirations. Reasons for this immobility include the social stigma associated with unsuccessful returns, a compensating motive for their heavy investment in the immigration process, a comparison of India and the United Kingdom in terms of objective success such as wages per hour, and the consideration of ultimate future happiness resulting from unhappy journeys in the present. In this regard, the present findings align with Sin’s (2016) explanations on the intersections between higher educational institutions and ethnicities in the United Kingdom. Furthermore, the present findings elaborate on and extend Sin’s explanation, indicating that the prospect of gaining cultural capital through UK higher education is a leading cause of Indian student immigrants’ involuntary immobility, which in turn causes a decline in positive feelings in their immigrant lives.

Carling and Schewel (2018) described that involuntary immobility is a severe state of immigration dynamics associated with sending countries. Several studies confirmed this argument by studying refugees and explaining that they become involuntarily immobile due to structural reasons such as border control (Lubkemann, 2008; Smets, 2019). However, the findings of the present study illustrate that involuntary immobility can develop even within host countries, disrupting the freedom of mobility among immigrants. Although immigrants have higher levels of aspirations, socioeconomic and financial challenges can foster their immobility, rendering them unable to effectively translate the facilities and credentials of the host country into positive outcomes. As a result, the findings imply that there is an incongruence between higher levels of aspirations, limited freedom of agency, and the ability to positively carve out future expectations in migrant lives.

While factors such as the fragility of aspirations and the uncertainties inherent in daily lives are readily apparent as contributors to decline in life satisfaction, the findings highlight that acculturative distress, social isolation, unmet career aspirations, challenges in the United Kingdom education system, and difficulties with visa pathways are prime factors affecting immigrants. Even though previous research has identified acculturative distress (Hendriks, 2015; Kóczán, 2016), the present study reveals that acculturative distress is linked to both existing and potential future uncertainties in immigrants’ lives. Social networks are a vital ingredient for immigrants’ happiness (Amit and Riss, 2014; Udayanga et al., 2023); however, participants in the study acknowledged that access to existing social networks is challenging, resulting in inadequate support from these networks. While they have access to certain social networks, such as student and Indian community groups, their ability to facilitate integration for immigrants in the United Kingdom context is relatively limited. The collapse of career aspirations, recognized by several studies as a reason for both objective and subjective well-being, is confirmed in the present study. It also indicates that the educational credentials of immigrants are less likely to be effectively translated into better labor market outcomes.

Moreover, Schmidt et al. (2023) found that bureaucratic processes can lead to the precarity of immigrants. The findings of the present study contribute to this argument, suggesting that declined subjective well-being is associated with the challenges immigrants face while navigating immigration bureaucracies, including the search for sponsorship to remain on visa pathways. Jacobs (2019) extends this claim in the United States context indicating that class of admission to the country, the time spend after the migration, and the legal channel they follow for permanent residency are closely connected. Furthermore, difficulties accessing healthcare and weather-related depression are potential drivers that can lead to a decline in life satisfaction. However, these factors are also recognized as impediments that can undermine the ability of highly educated immigrants to effectively exercise agency in navigating the challenges associated with immigrant life.

The present study questioned the widely held belief that migration can first increase subjective well-being and, then the possibility of decreasing subjective well-being over time. It suggests that the migration channel is a vital determinant that can shape the experiences of immigrants, impacting their life satisfaction. For those who immigrated primarily for higher education and then to settle in the country, the disabling effect (the limited ability of agency to navigate challenges in the host society) can hamper well-being outcomes. During the three-step migration process, immigrants do not show increased subjective well-being even at the beginning of migration, and ‘migration life’ has become an additional burden, in addition to several other challenges they encounter. The study findings mainly implied that, despite the initial belief that migration would enhance life satisfaction, Indian student immigrants have become involuntarily immobile in the United Kingdom, unable to effectively leverage their higher education to establish aspirations. The incongruency between what was expected and what is presently being experienced creates a sense of regret among participants in the present study, leading them to think that life would have been better in their home country. All of these suggest that migration comes at a cost, along with several other challenges, particularly for those who have moved first to learn, secondly to earn, and then to settle in.

## Data availability statement

The raw data supporting the conclusions of this article will not be available for public use, due to ethical concerns. Further inquiries can be directed to the corresponding author/s.

## Ethics statement

The study was approved by the research ethics committee of the Faculty of Humanities and Social Sciences, University of Ruhuna, Sri Lanka. The studies were conducted in accordance with the local legislation and institutional requirements. The participants provided their written informed consent to participate in this study.

## Author contributions

SU: Conceptualization, Data curation, Formal analysis, Funding acquisition, Investigation, Methodology, Project administration, Resources, Software, Supervision, Validation, Visualization, Writing – original draft, Writing – review & editing.

## Footnotes

^1^The Russell Group is a prestigious association of 24 leading public research universities in the United Kingdom. These institutions are renowned for their commitment to research excellence, high academic standards, and significant influence on education and policy.

^2^Any immigrants who want to stay in the United Kingdom for a long period of time on a work visa must obtain sponsorship from an employer willing to host them. However, in order to do so, these employers must obtain the necessary permits from the immigration bureaucracy. But, most of the time, these employers lack Home Office registration, which prevents them from providing a sponsorship letter to immigrants.
